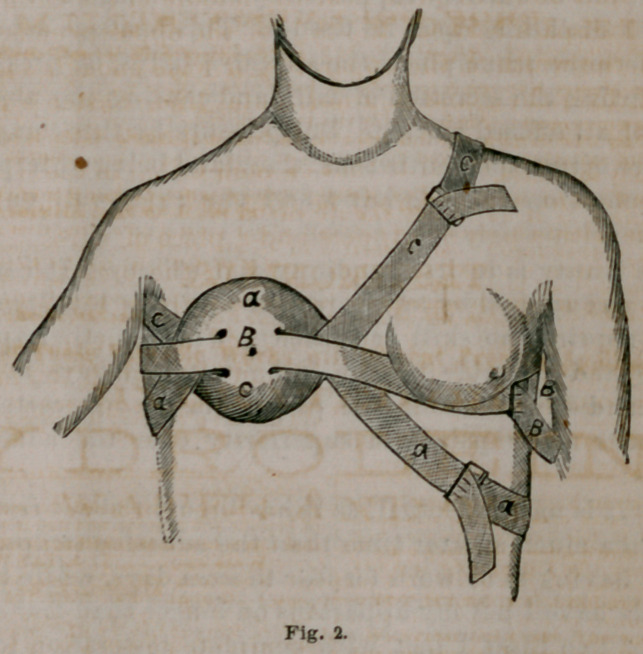# Abortive Treatment of Mammary Abscesses and the Cure of Fissured Nipples by Means of a New and Effectual Compress

**Published:** 1882-10

**Authors:** George H. Noble

**Affiliations:** Atlanta, Ga.


### ABORTIVE TREATMENT OF MAMMARY ABSCESSES,

AND

### THE CURE OF FISSURED NIPPLES BY MEANS OF A NEW AND EFFECTUAL COMPRESS.

By GEO. H. NOBLE, M.D., Atlanta, Ga.

A little more than eighteen months ago I found a patient, a few days after confinement, strongly threatened with a mammary abscess. Indeed there was considerable inflammation of the left breast, it being greatly swollen, indurated and sensitive to the touch. An abscess seemed imminent, and the pain and distress called for immediate relief.

Having no adhesive plaster with me, and being quite distant from where it could be procured, my time would not allow me to wait until a messenger could be sent for it; so I accordingly asked for a piece of cotton goods wide enough to cover the gland and sufficiently long to little more than encircle the chest. This I split up at each end into three tails of equal width, extending the rents down very near to the center of the cloth, thus leaving a body, as it were, its entire width, with the three tails attached on either side, as seen in Fig. 1.

Of this I made a compress, as will subsequently be shown, and brought firm pressure to bear upon the entire breast, which effected the most surprising, yet favorable results.

After smoothly and evenly applying my compress, I left the patient, only to return twenty-four hours later, when I found that the gland had lost half of its increased size, the hardness was very much lessened, and the soreness greatly ameliorated. The improvement was so great that it seemed scarcely necessary to continue the application. I however, drew the apparatus tighter and left it until the following day, when I found that the breast had returned to its *normal size*, and that the hardness and soreness were no longer present. Even upon the very first application she expressed herself as being very greatly relieved of the pain, weight, and heavy dragging sensation. The improvement was so very rapid that at the end of forty-eight hours all unpleasantness had subsided, and the further wearing of the apparatus was deemed unnecessary. This, I consider, was a remarkably rapid restoration in such an urgent case.

You will remember that the best means of treatment heretofore at our command, (adhesive plaster), has not established resolution in so short a time. This is one of the points of superiority that I advance over the plaster.

Perceiving this and other advantages, hearing no complaint of discomfort on the part of my patient, and being greatly encouraged by the success in the preceding case, I determined to try its efficacy in such other cases as might come before me. I did so, and with the most gratifying results; so I now present it to the profession that it may have the test of all unprejudiced professional minds. Yet I ask that they will not render a decision until they have given it a fair trial and have observed all the points as to accuracy of application that I shall give.

The compress may be made of almost any ordinary cotton goods, such as sheeting, shirting etc.; and these are usually at hand either in one form or another.

Measure over the nipple in a vertical direction from base to base of the gland, and take about four-fifths of this for the width of the bandage—if the breasts are very large it may require a little more. Next cut the cloth long enough to little more than encircle the chest over the affected mammæ. Divide each end into three equal parts and split down to quite near the middle of the cloth, leaving the space between the tails of the opposite ends about half the width of the apparatus, or wide enough to about half cover the gland in a horizontal line. Fasten a buckle on the tails at one end of the compress, the width of which must be equal to that of each tail. In case buckles of sufficient width cannot be obtained, they can easily be made with a pair of nippers and a piece of wire.

If there should be much milk in the breast, it is best to make a small aperture in the centre of the compress, that the nipple may protrude and allow the slow and steady escape of that fluid. I find that when the milk is permitted to escape in this manner the reduction in size is much more readily effected.

Fig. 1.

The margins of the compress should not be hemmed, as the thick edges will cause some inconvenience where they press upon the inflamed organ; neither will they lay as close and smoothly as the raw borders. In the first case or two that I applied the compress I tied the tails, but as the knots caused some little annoyance, I substituted the buckles. The tails might be left one-third longer on one end than upon the other, so that the buckles will be brought around to the anterior part of the chest; this, however, is a matter of no great importance, as a small pad may separate them from the chest walls.

The application is simple and easy. First have an assistant to elevate the breast to its utmost extent, and then place the bandage underneath it so that the lowest pair of tails (*c.c.* Fig. 2) will lay upon the under side and base of the gland close to the chest wall. Draw the tails (*c.c.*) upwards, the one under the axilla of the affected side, and the other over the shoulder of the side opposite to the inflamed organ; draw tightly and buckle so as to take the entire weight of the breast.

Fig. 2.

The next pair of tails (*a.a.*) should be turned over the gland and drawn downward, so as to force the breast down against the preceding pair of tails, and buckled around the lower part of the chest. Next, the middle tails (*b.b.*) should be carried directly around the chest and fastened in a similar manner; but the tail on the side of the sound breast should go under and not over that organ.

Now, begin in the same order and tighten each pair of tails until you have brought firm pressure *equally* upon the whole organ. If the gland should tend to protrude between the tails, a few stitches with a needle and thread or a pin may be used to approximate the edges and to retain the protruding parts. Fig. 2 shows compress applied.

As the tumefaction subsides, it will be necessary to tighten the apparatus to keep up a continuous pressure. This the nurse or patient herself may do, but care must be taken that there may be no constriction or greater tension on one part than upon another.

The bandage must look smooth and round; the breast must be drawn up higher than its fellow, the healthy gland, and be entirely supported by the bandage, so that no weight shall drag upon its tissues. This bandage acts upon the same principle as the adhesive strips, by pressure, preventing the secretion of milk and causing the absorption of all effused matter. But pressure by this compress is more direct and continuous, the gland being interposed between it on the one hand and the chest-wall on the other.

Its beauty is in its simplicity and efficacy. It can be made and applied by any person of ordinary intelligence, as it requires no skill or practice; while another appreciable and valuable feature is that it is always at our command—even among the poorer classes. It certainly possesses many points of superiority over the adhesive straps:

I. It is more effectual, as it has brought about restoration in a much shorter time than the adhesive straps, the latter having to be worn for *four* to *seven* days, while in *no instance* have I left my apparatus on longer than *four days*, and in two cases I met with complete restoration in *two* days. (See appended reports.)

II. It does not give the uneasiness and discomfort that the stiff, hard plaster occasions, as the cloth adapts itself to the gland, and allows a certain degree of movement that the plaster does not permit.

III. We get equal pressure on all parts of the gland by close adaptation of the cloth to it, and by tightening and retightening the different tails until we have brought the cloth in close contact with *all parts* of the breast. This cannot be done with the adhesive plaster, which, if it should not be properly put on, must be removed and another applied.

IV. We can determine the amount of pressure we put upon the gland by the force with which we draw upon the tails.

V. It affords great advantage over the plaster in enabling us to tighten, from time to time, without removing the dressing, thereby bringing increased pressure to bear upon the breast as it, from the subsidence of the tumefaction, shrinks away from the apparatus. With the plaster we are much inconvenienced by the necessity of reapplying it as the parts decrease in size.

VI. It is cooler, more comfortable and easy.

VII. As above stated, it is always at our command.

VIII. The cloth possesses a certain degree of elasticity, which causes the compress to contract upon the breast as the tumefaction subsides.

IX. The patient or nurse can keep the necessary tension on the organ by tightening the apparatus, while the doctor has to apply the adhesive strips each time.

X. It admits of the use of any medicated application that may be fancied, though such treatment is unnecessary.

XI. It is an estimable apparatus for checking the secretion of milk when the child is suddenly withdrawn from the breast.

XII. It is an adjunct to the weaning of a child. Strap the breast and let the child try it. When he finds that there is no milk to be had he will turn to another source for nourishment.

XIII. I believe it to be the best means of treating fissured nipples. Apply the compress, remove the child from the breast and in a few days the fissure will be well when the child may be returned.

I subjoin reports of a few cases in which the compress has been employed.

N. B., aged twenty-four years, on third day after her second confinement she was found to be suffering considerably with her left breast. It was greatly swollen, indurated and sensitive to the touch.

An abscess seemed imminent, but fortunately I applied this many tailed bandage or compress with the most remarkable results. In this case I intended merely to make temporary use of it and to apply the adhesive strips on the following day; but upon my return I was so greatly astonished and so agreeably surprised that I determined to continue the treatment with this apparatus.

I found that the tumefaction had more than half disappeared, the induration was almost entirely removed, while no soreness was found to be present.

As I have just stated, my great surprise at the results of the temporary bandage caused me to make further use of it; so I tightened it and again brought firm pressure to bear upon the breast for twenty-four hours more. This proved sufficient to effect complete restoration of the organ to its natural or normal condition; the child being withheld from the breast for some three or four days only.

This abortion of the mammary abscess in forty-eight hours is the quickest that I know of, save in one other instance the report of which I here append:

II. Mrs. W. M., aged twenty-six years. The condition of this patient was very similar to the preceding. The symptoms were very urgent and distressing. The presence of pus seemed almost conclusive. The compress was the sole means of treatment resorted to, and most happily did it act.

The patient expressed great relief immediately upon its application, as it relieved the weight and heavy dragging sensation.

The bandage was applied with very great tension and I left to return the following day, when I found the symptoms were very much ameliorated.

I again increased the tension and left it for a similar period of time.

Upon the third day recovery was complete. The nipple did not protude and the milk did not escape as in the foregoing case, so the resolution was necessarily prolonged to a little greater time than in that of case I.

III. Mrs. B. B., aged thirty years, mammitis. Both breasts enlarged to twice their natural size, very sore and hard. She loses sleep from pain.

Compress applied May 16th, 1881, to both glands, nipples allowed to protude and milk to slowly escape.

May 17th. The glands have been reduced until only one-third larger than their natural size.

I again brought pressure to bear upon the breasts and left the apparatus to complete its work in the resolution.

May 18th. All the trouble has completely subsided leaving the mammæ in such a good condition that the bandage has been removed.

This case showed no tendency to a recurrence of the disorder, so the resolution was effected in three days.

IV. Mrs. J. A., aged forty-four years, miscarried at four months from placenta prævia. Mammary abscess strongly threatened in left breast, could not bear the slightest manipulation of that organ. The gland measured eighteen inches in circumference. The induration was mainly on the inner or right side of the gland. Compress brought down the measurement in twenty-four hours to fourteen inches. Induration greatly lessened. Compress was tightened as in the other cases. On the following day the breast's circumferential measurement was normal, being twelve inches only. Restoration in two days.

V. Mrs. J. F., aged twenty-eight years, miscarried at about four and a half months. Both breasts became in flamed and greatly enlarged, the right and left measuring respectively sixteen, and seventeen and a half inches. Induration through the entire mammæ, soreness considerable.

Compress applied July 11th, 1882.

July 12th. Each breast measures four inches less, and is correspondingly improved as to the other symptoms.

July 13th. The reduction in size does not appear to be so great, being one inch only to each gland. The compress was drawn rather more firm and nipples allowed to protrude.

July 14th. The two glands have resumed their usual size, now measuring fourteen inches. All other symptoms have so rapidly disappeared that the compress was removed, and the case set down as a cure in four days, the longest period required by any one case, either double or single.

It will be seen by the foregoing reports that two were cured in two days, two in three, and the fifth case in four days.

This shows better results than the adhesive strips, as it usually requires from four to seven days in an ordinary case. The compress is more comfortable, convenient, easy of application, and I am satisfied all that is necessary for any one to convince himself that it is the best means, as yet, at our command for the treatment of mammitis and for aborting mammary abscess is to observe all the details of its application, fit it accurately and comfortably, apply all the pressure the patient can bear, and if a fair trial is then given it, it will, I feel confident, receive an endorsement.

Since writing the above I have applied my compress in a case threatened with mammary abscess in both breasts with fissure of both nipples. I applied the compress to one gland and removed the child from that organ. In three days the fissure was well, and all other symptoms were gone.

In the meantime the breast least affected was treated with belladonna, camphor and some other medicaments, which seemed to hold the symptoms in check until the other breast was cured, and the compress removed and applied to its fellow. Two days were sufficient to cure the second fissure, and to relieve the pain and swelling.

In a second and very similar case the bandage was applied to both breasts and the child removed from its mother. The fissures were cured in a day or two, the compress was removed, and the secretion of milk again commenced, and the child was returned to its mother.

In a third and similar case I applied the compress for a medical friend, and instructed him to continue the treatment as in the second case of fissure. He reported a like improvement.

From the foregoing it will be seen that the method I propose surpasses the adhesive plaster, not only in the treatment of mammitis and in securing the abortion of mammary abscesses, but it is also the best means of treating fissured nipples. Hence, it is hoped that from its use great good will accrue.

## Figures and Tables

**Fig. 1. f1:**
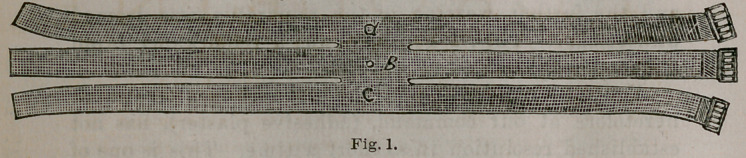


**Fig. 2. f2:**